# Analysis of sex-specific risk factors and clinical outcomes in COVID-19

**DOI:** 10.1038/s43856-021-00006-2

**Published:** 2021-06-30

**Authors:** Tomi Jun, Sharon Nirenberg, Tziopora Weinberger, Navya Sharma, Elisabet Pujadas, Carlos Cordon-Cardo, Patricia Kovatch, Kuan-lin Huang

**Affiliations:** 1 Division of Hematology and Medical Oncology, Tisch Cancer Institute, Icahn School of Medicine at Mount Sinai, New York, NY USA; 2 Scientific Computing, Icahn School of Medicine at Mount Sinai, New York, NY USA; 3 Department of Genetics and Genomic Sciences, Center for Transformative Disease Modeling, Tisch Cancer Institute, Icahn Institute for Data Science and Genomic Technology, Icahn School of Medicine at Mount Sinai, New York, NY USA; 4 University of California, Santa Barbara, CA USA; 5 Department of Pathology, Molecular and Cell-Based Medicine, Icahn School of Medicine at Mount Sinai, New York, NY USA

**Keywords:** Viral infection, Public health, Epidemiology

## Abstract

**Background:**

Sex has consistently been shown to affect COVID-19 mortality, but it remains unclear how each sex’s clinical outcome may be distinctively shaped by risk factors.

**Methods:**

We studied a primary cohort of 4930 patients hospitalized with COVID-19 in a single healthcare system in New York City from the start of the pandemic till August 5, 2020, and a validation cohort of 1645 patients hospitalized with COVID-19 in the same healthcare system from August 5, 2020, to January 13, 2021.

**Results:**

Here we show that male sex was independently associated with in-hospital mortality, intubation, and ICU care after adjusting for demographics and comorbidities. Using interaction analysis and sex-stratified models, we found that hypoxia interacted with sex to preferentially increase women’s mortality risk while obesity interacted with sex to preferentially increase women’s risk of intubation and intensive care in our primary cohort. In the validation cohort, we observed that male sex remained an independent risk factor for mortality, but sex-specific interactions were not replicated.

**Conclusions:**

We conducted a comprehensive sex-stratified analysis of a large cohort of hospitalized COVID-19 patients, highlighting clinical factors that may contribute to sex differences in the outcome of COVID-19.

## Plain Language Summary

Men are at higher risk of death from COVID-19 than women, but the underlying reasons are not fully understood. We examined the medical data of men and women hospitalized with COVID-19 in New York City to determine whether there were factors which raised the risk of death or requiring intensive care more for one sex rather than the other. We observed that men hospitalized with COVID-19 had a higher risk of death than women when other factors taken into account. Some conditions, like low oxygen levels and obesity, appeared to be associated with worse outcomes in women compared to men early in the pandemic but further studies will be necessary for confirmation. These findings highlight groups of men and women who may be at increased risk of severe COVID-19.

## Abstract (web summary)

Jun et al. evaluate sex-stratified clinical outcomes in two cohorts of patients hospitalized with COVID-19 in New York. While male sex risk is a risk factor for poor outcome in both cohorts – one from earlier and one from later on in the pandemic – some of the sex-specific risk factors observed initially are not observed later on.

## Introduction

Reports of the COVID-19 pandemic from around the world have described more severe disease and worse outcomes among men^1–7^. Men with COVID-19 appear to preferentially require hospitalization and intensive care^2,8,9^. For example, one large Italian case series reported that 82% of COVID-19 patients requiring intensive care were men^10^. Case fatality rates are also higher among men; a nationwide analysis from China reported a case fatality rate of 2.8% for men, compared to 1.7% for women^4^. Male sex has been identified as a risk factor for mortality in several studies of hospitalized COVID-19 patients^11–15^.

These observations have been variously attributed to underlying comorbidities among men, hormonal factors, or immune differences between men and women^16–19^. Some comorbidities associated with worse COVID-19 outcomes may be more common among men, though published studies have not provided sex-disaggregated data^2,20,21^. An Italian study observed that among prostate cancer patients, those on androgen deprivation therapy had better outcomes than those who were not, suggesting a sex hormonal contribution to COVID-19 mortality^22^. In addition, there are sex-specific differences in expression of the ACE2 and TMPRSS2 proteins, which facilitate the entry of the SARS-CoV-2 virion into cells^23,24^. Understanding how risk factors of severe disease differ between sexes can improve clinical risk assessment and shed new biological insights into disease etiology.

Given the sex difference in COVID-19 outcomes, we hypothesized that clinical risk factors for mortality might show sex-specific effects, which remain largely uncharacterized. Although multiple prior studies have included multivariable regression models adjusting for sex, they have not explored the possibility of interactions between sex and other predictors, nor have they examined differences in clinical course between men and women admitted with COVID-19.

To fill this knowledge gap, we conduct a sex-specific analysis of clinical data from 4930 COVID-19 patients hospitalized in New York City from the start of the pandemic to August 5, 2020. We confirm the association between male sex and adverse COVID-19 outcomes in this primary cohort and in a validation cohort of 1645 COVID-19 patients hospitalized from August 5, 2020, to January 13, 2021. We also explore sex-specific risk factors of in-hospital mortality, intubation, and intensive care.

## Methods

### Study setting

Mount Sinai Health System is a large hospital system in the New York City area, comprising 8 hospitals and more than 410 ambulatory practice locations. Our analysis focused on patients who were admitted to five hospitals: The Mount Sinai Hospital (1134 beds), Mount Sinai West (514 beds), and Mount Sinai Morningside (495 beds) in Manhattan; Mount Sinai Brooklyn (212 beds) in Brooklyn; and Mount Sinai Queens (235 beds) in Queens.

### Data sources

Data were derived from clinical records from Mount Sinai facilities using the Epic electronic health record (Epic Systems, Verona, WI). Data were directly extracted from Epic’s Clarity and Caboodle servers. In the setting of the COVID-19 pandemic, the Mount Sinai Data Warehouse (MSDW) developed and released a de-identified dataset encompassing all COVID-19 related patient encounters within the Mount Sinai system, accompanied by selected demographics, comorbidities, vital signs, medications, and lab values. As part of de-identification, all patients over the age of 89 had their age set to 90. Updated versions of the dataset have been released on a weekly schedule. For this study, we used the dataset released on August 5, 2020.

### Patient population and definitions

The MSDW dataset captured 224,018 inpatient and outpatient encounters involving 144,518 distinct patients at a Mount Sinai facility with any of the following: a COVID-19 related encounter diagnosis, a COVID-19 related visit type, a SARS-CoV-2 lab order, a SARS-CoV-2 lab result, or a SARS-CoV-2 lab test result from the New York State Department of Health’s Wadsworth laboratory up to August 5, 2020. Of these, 75,996 patients had at least one COVID-19 test and 12,347 tested positive. COVID-19 positivity was defined as a positive or presumptive positive result from a nucleic acid-based test to detect SARS-CoV-2 in nasopharyngeal or oropharyngeal swab specimens.

We limited our analysis to 4930 adult patients (at least 18 years old) who were admitted to the hospital and had a positive or presumptive positive SARS-CoV-2 test within 2 days of admission. Race and ethnicity were self-reported. Comorbidity status was assessed cross-sectionally using each patient’s problem list in the electronic medical record as of their hospital encounter. The problem list consists of diagnoses linked to International Classification of Disease codes; it is updated by medical providers during clinical encounters (inpatient and outpatient) and reflects ongoing medical issues. Obesity was based on body mass index (BMI) ≥ 30 kg/m^2^ or a pre-existing diagnosis in the medical record if BMI was not available.

The dataset captured inpatient medication exposures for a pre-selected list of drugs, including COVID-19 therapeutics such as remdesivir, steroids, and dexamethasone. Of note, medications administered in the context of clinical trials were not captured in this dataset.

Initial vital signs were the first vital signs documented for the encounter. Fever was defined as temperature ≥100.4 °F; tachycardia was defined as heart rate >100 beats per minute; tachypnea was defined as respiratory rate >25 breaths per minute; hypotension was defined as systolic blood pressure <90 mmHg or mean arterial pressure <65 mmHg; hypoxia was defined as oxygen saturation <92%.

We defined initial labs as the first lab value within 24 h of the start of the encounter. A subset of patients had baseline serum interleukin-1-beta (IL-1B), interleukin-6 (IL-6), interleukin-8 (IL-8), and tumor necrosis factor-alpha (TNF-a) values obtained as part of a study, which enrolled COVID-19 patients hospitalized between March 21 and April 28, 2020^25,26^.

SARS-CoV-2 viral loads from nasopharyngeal swabs were measured in a randomly selected cohort of 1146 MSHS patients hospitalized between March 13 and May 4, 2020, using quantitative RT-PCR^27^. Direct overlap with our de-identified dataset was not feasible, but this random sample was drawn from the same population as our cohort and is likely representative.

### Outcomes

The primary outcome was death from any cause during admission. The primary outcome was known for 99.5% of patients; only 23 patients had missing data. Secondary outcomes were mechanical ventilation during admission and intensive care unit (ICU)-level care (defined as admission to an ICU or mechanical ventilation) during admission.

### Logistic regression analysis

Univariable and multivariable logistic regression were used to identify factors associated with each outcome. To identify sex-specific effects of risk factors, subgroup analysis was performed using a multivariable model including an interaction term between sex and the subgroup variable. This was performed for each subgroup variable to identify significant sex interactions. We further used sex-stratified multivariable models to estimate the magnitude of effect of each covariate within each sex cohort.

Predictors analyzed included demographic factors, comorbidity status, initial vital signs, treatment facility location (Manhattan vs. Brooklyn/Queens), and time period (prior to April 13, April 14 to June 2, June 2 to August 5). There was minimal clustering of the primary outcome by treatment site (ICC (ρ) = 0.021). Covariates were chosen a priori based on prior reports and low missingness. The covariates included in the final models were age (as a categorical variable), sex, race/ethnicity, treatment location, treatment time period, baseline hypoxia, and the following comorbidities: hypertension, diabetes, coronary artery disease, heart failure, atrial fibrillation, chronic kidney disease, chronic obstructive pulmonary disease (COPD) or asthma, obesity, cancer, chronic liver disease. We report the odds ratios derived from the coefficients of each model, along with the Wald-type confidence interval and *p* values.

### Statistics

Patient characteristics and baseline vitals and labs were described using medians and ranges for continuous variables, and proportions for categorical variables. Continuous variables were compared using the Wilcoxon rank-sum test, and categorical variables were compared using Fisher’s exact test or the chi-squared test, as appropriate. All statistical analyses and data visualizations were carried out using R 4.0.0 (The R foundation, Vienna, Austria), along with the *tidyverse*, *ggpubr*, *forestplot*, and *Hmisc* packages. Statistical significance was defined as a two-tailed *p* < 0.05 unless otherwise specified.

### Study approval

The MSDW project (GCO# 12-0361) was reviewed and approved by the Institutional Review Board (IRB) of the Mount Sinai School of Medicine. The MSDW serves as a repository for data from the electronic medical record which can be used for research. The IRB has determined that the MSDW research involves no greater than minimal risk and approved the waiver for informed consent. A de-identified COVID-19 dataset was generated by the MSDW team for use by the Mount Sinai research community. The present study used this de-identified dataset for the rest of the analyses.

### Reporting Summary

Further information on research design is available in the Nature Research Reporting Summary linked to this article.

## Results

The study cohort comprised 4930 COVID-19 patients hospitalized within the Mount Sinai Health System in New York City, of whom 2757 (55.9%) were men and 2173 (44.1%) were women. The majority (65.4%) of patients were treated in Manhattan facilities, which had larger bed capacities than facilities in Brooklyn or Queens. Most of the patients in the cohort (65.3%) were admitted before April 13, 2020, which corresponded to the peak of COVID-19 admissions within the hospital system.

### Demographics and comorbidities

Women in the cohort were older overall. A greater proportion of women were 75 or older (36.6% vs. 26.5%, Fisher’s exact *p* = 2.8 × 10^−14^) and the median age among women was 68 (IQR 55–80) compared to 65 (IQR 53–75, Wilcoxon *p* = 1.1 × 10^−10^) among men. Women in the cohort were more likely to have hypertension (38% vs. 34.9%, %, Fisher’s exact *p* = 0.03), COPD/asthma (10.9% vs. 7.2%, %, Fisher’s exact *p* = 6.6 × 10^−6^), and to be obese (38.9% vs. 27.9%, %, Fisher’s exact *p* = 3.9 × 10^−16^). Men in the cohort were more likely to be a current or former smoker (31.1% vs. 20.6%, %, Fisher’s exact *p* = 4.2 × 10^−24^) and to have chronic kidney disease (13.1% vs. 11%, %, Fisher’s exact *p* = 0.03), cancer (8.1% vs. 6.6%, %, Fisher’s exact *p* = 0.04), chronic liver disease (3.9% vs. 2.6%, %, Fisher’s exact *p* = 0.02), and HIV (2.4% vs. 0.9%, %, Fisher’s exact *p* = 4.6 × 10^−5^). Additional clinical features are presented in Table 1.

**Table 1:** Baseline clinical characteristics, by sex.

Variable	Male (*N* = 2757)	Female (*N* = 2173)	*p* value	Overall (*N* = 4930)
Age (yrs)	65 (53–75)	68 (55–80)	<0.001	66 (54–78)
Age < 55	758 (27.5%)	527 (24.3%)	0.01	1285 (26.1%)
Age 55–64	617 (22.4%)	359 (16.5%)	<0.001	976 (19.8%)
Age 65-74	651 (23.6%)	491 (22.6%)	0.4	1142 (23.2%)
Age ≥ 75	731 (26.5%)	796 (36.6%)	<0.001	1527 (31%)
Asian	137 (5%)	89 (4.1%)	0.2	226 (4.6%)
Hispanic	780 (28.3%)	613 (28.2%)	0.9	1393 (28.3%)
Non-Hispanic Black	674 (24.4%)	630 (29%)	<0.001	1304 (26.5%)
Non-Hispanic White	637 (23.1%)	534 (24.6%)	0.3	1171 (23.8%)
Other race/ethnicity	443 (16.1%)	247 (11.4%)	<0.001	690 (14%)
Manhattan facility	1741 (63.1%)	1481 (68.2%)	<0.001	3222 (65.4%)
Before April 13	1860 (67.5%)	1358 (62.5%)	<0.001	3218 (65.3%)
April 13 to June 2	782 (28.4%)	717 (33%)	<0.001	1499 (30.4%)
June 2 to August 5	115 (4.2%)	98 (4.5%)	0.6	213 (4.3%)
Current/former smoker	857 (31.1%)	448 (20.6%)	<0.001	1305 (26.5%)
Body mass index (kg/m^2^)	27.0 (23.7–31.1)	28.4 (24.3–33.9)	<0.001	27.5 (23.9–32.3)
Hypertension	961 (34.9%)	825 (38%)	0.03	1786 (36.2%)
Diabetes	653 (23.7%)	543 (25%)	0.3	1196 (24.3%)
Coronary artery disease	403 (14.6%)	282 (13%)	0.1	685 (13.9%)
Heart failure	220 (8%)	164 (7.5%)	0.6	384 (7.8%)
Atrial fibrillation	222 (8.1%)	148 (6.8%)	0.1	370 (7.5%)
Chronic kidney disease	360 (13.1%)	239 (11%)	0.03	599 (12.2%)
COPD/asthma	199 (7.2%)	237 (10.9%)	<0.001	436 (8.8%)
Obesity	770 (27.9%)	846 (38.9%)	<0.001	1616 (32.8%)
Cancer	224 (8.1%)	143 (6.6%)	0.04	367 (7.4%)
Chronic liver disease	107 (3.9%)	57 (2.6%)	0.02	164 (3.3%)
HIV	67 (2.4%)	20 (0.9%)	<0.001	87 (1.8%)
Initial vital signs				
Temperature (°F)	98.7 (98–100)	98.6 (98–99.8)	0.001	98.6 (98–99.9)
Fever	586 (21.3%)	391 (18%)	0.005	977 (19.8%)
Heart rate (bpm)	97 (84–111)	93 (82–108)	<0.001	96 (83–110)
Tachycardia	1187 (43.1%)	787 (36.2%)	<0.001	1974 (40%)
Systolic blood pressure (mmHg)	130 (116–147)	127 (113–143)	<0.001	129 (115–145)
Hypotension	92 (3.3%)	84 (3.9%)	0.4	176 (3.6%)
Respiratory rate (bpm)	20 (18–22)	20 (18–22)	<0.001	20 (18–22)
Tachypnea	422 (15.3%)	300 (13.8%)	0.1	722 (14.6%)
Oxygen saturation (%)	95 (92–98)	96 (93–98)	<0.001	95 (92–98)
Oxygen sat. <92%	670 (24.3%)	440 (20.2%)	<0.001	1110 (22.5%)

Fever: temperature ≥100.4 °F; tachycardia: heart rate >100 beats/min; tachypnea: respiratory rate >25 breaths/min; hypotension: systolic blood pressure <90 mmHg or mean arterial pressure <65 mmHg.

### Vital signs and laboratory values at presentation

At presentation, men were more likely to be febrile (21.3% vs. 18%, Fisher’s exact *p* = 0.005), tachycardic (43.1% vs. 36.2%, Fisher’s exact *p* = 1.3 × 10^−6^), and hypoxic (24.3% vs. 20.2%, Fisher’s exact *p* = 8.6 × 10^−4^). Men and women had similar white blood cell counts (7.9 vs. 7.6 K/μL, Wilcoxon *p* = 0.1), but men had lower lymphocyte counts (0.87 vs. 1.05 K/μL, Wilcoxon *p* = 1.4 × 10^−26^). Among inflammatory markers, C-reactive protein was higher in men than women at baseline (125.3 vs. 88.4 mg/L, Wilcoxon *p* = 3.4 × 10^−21^), whereas ferritin was more elevated (relative to the upper limit of normal [ULN] for each sex) in women than in men (3.06 vs. 2.35 xULN, Wilcoxon *p* = 5.4 × 10^−8^).

A subset of patients had IL-1B, IL-6, IL-8, and TNF-a measured from serum at baseline. Of these cytokines, only IL-6 was significantly different between men and women (78.7 vs. 57.9 pg/mL, Wilcoxon *p* = 7.2 × 10^−5^). Additional laboratory values are presented in Supplemental Table 1.

We examined SARS-CoV-2 viral load by sex using a randomly selected cohort of 1146 MSHS patients hospitalized between March 13 and May 4, 2020, previously described by Pujadas et al.^27^. This cohort consisted of 494 (43.1%) female and 651 (56.9%) male patients, with average log_10_ viral loads of 5.61 and 5.51 copies/mL, respectively (Welch *t*-test *p* = 0.53) (Supplemental Fig. 1).

### Treatment and outcomes

The most common treatments administered in the cohort were as follows: hydroxychloroquine (*N* = 3156, 64%), azithromycin (*N* = 3070, 62.3%), and steroids (*N* = 1343, 27.2%) including methylprednisolone (*N* = 1067, 21.6%), prednisone (*N* = 386, 7.8%), dexamethasone (*N* = 164, 3.3%), and hydrocortisone (*N* = 15, 0.3%) (Supplemental Tables 2 and 3). Overall, men were more likely to receive hydroxychloroquine (67.5% vs. 59.5%, Fisher’s exact *p* = 7.8 × 10^−9^), azithromycin (65.5% vs. 58.2%, Fisher’s exact *p* = 1.9 × 10^−7^), and steroids (29.1% vs. 24.9%, Fisher’s exact *p* = 0.001). However, there were no significant differences in the use of medications between men and women requiring ICU-level care (Supplemental Tables 2 and 3).

Treatment patterns in the cohort changed over time. The proportion of patients receiving remdesivir and dexamethasone increased over the time periods of pre-April, April–June, and June–August (remdesivir: 1.6% vs. 4.9% vs. 6.1%, chi-squared *p* = 1.4 × 10^−11^; dexamethasone: 2.8% vs. 3.1% vs. 12.7%; chi-squared *p* = 6.0 × 10^−14^), whereas the receipt of hydroxychloroquine and azithromycin decreased over the same period (hydroxychloroquine: 78.6% vs. 41.8% vs. 0%; azithromycin: 72.8% vs. 46.4% vs. 15%; chi-squared *p* < 2.2 × 10^−16^ for both).

There were 1198 in-hospital deaths (24.3%). The rate of in-hospital mortality was 25.2% in men and 23.1% in women (Fisher’s exact *p* = 0.09). Among the patients who died, the median time from admission to death was 7.1 days (IQR 3.2–13). There was no significant difference in the median length of time from admission to death between men (7.3 days, IQR 3.3–12.4) and women (6.2 days, IQR 2.9–13.2; Wilcoxon *p* = 0.24). Among the patients who did not die, the median length of admission was 6 days (IQR 3–11) and men spent longer in the hospital (median 6.2 days, IQR 3.3–10.8) than women (median 5.7 days, IQR 2.8–10.2; Wilcoxon *p* = 0.004).

1176 patients (23.9%) received ICU-level care during their admissions; 737 patients (14.9%) received invasive ventilation. More men than women received ICU-level care (26.7% vs. 20.3%, Fisher’s exact *p* = 1.8 × 10^−7^) and invasive ventilation (16.4% vs. 13.2%, Fisher’s exact *p* = 0.002). The mortality rate among ICU patients was 55.7%; among intubated patients, it was 68.5%. There were no differences in mortality between men and women among ICU (56.6% vs. 54.1%, Fisher’s exact *p* = 0.43) or intubated (70.1% vs. 66.1%, Fisher’s exact *p* = 0.25) patients. Among those who were intubated, the median number of days from admission to intubation was 1.3 (IQR −0.3 to 5.4). There was no significant difference in the median time to intubation between men and women (1.4 vs. 1.3 days, Wilcoxon *p* = 0.69).

There was no significant difference in the proportion of men and women developing acute kidney injury (7% vs 6%, Fisher’s exact *p* = 0.2) or venous thromboembolism (1% vs. 0.7%, Fisher’s exact *p* = 0.3) during their hospitalizations.

### Sex-specific predictors of hospitalization outcomes

In multivariable logistic regression models adjusting for demographics, comorbidities, admission time period, and baseline oxygen saturation, male sex was an independent predictor of in-hospital mortality (OR 1.24, 95% CI 1.06–1.44), intubation (OR 1.22, 95% CI 1.03–1.46), and ICU care (OR 1.37, 95% CI 1.19–1.59) (Supplemental Fig. 2 and Supplemental Table 4). Other predictors of mortality included obesity (OR 1.38, 95% CI 1.17–1.62), hypertension (OR 0.81, 95% CI 0.68–0.97), chronic kidney disease (OR 1.16, 95% CI 1.08–1.72), and older age (age ≥ 74 vs. 55–64 OR 3.35, 95% CI 2.71–41.5). Interestingly, while mortality increased with age, the youngest and oldest age groups were associated with less intubation (age < 55 OR 0.61, 95% CI 0.47–0.79; age ≥ 75 OR 1.22, 95% CI 1.03–1.46) and less ICU-level care (age < 55 OR 0.76, 95% CI 0.61–0.94; age ≥ 75 OR 0.65, 95% CI 0.52–0.8), compared to those aged 55–64 (Supplemental Fig. 2).

To compare predictors of mortality, intubation, and ICU care between men and women, we first performed subgroup analysis to identify significant interactions between sex and other covariates. Then, we used sex-stratified regression models to estimate the effect sizes of the individual predictors in each sex.

**Fig. 1:**
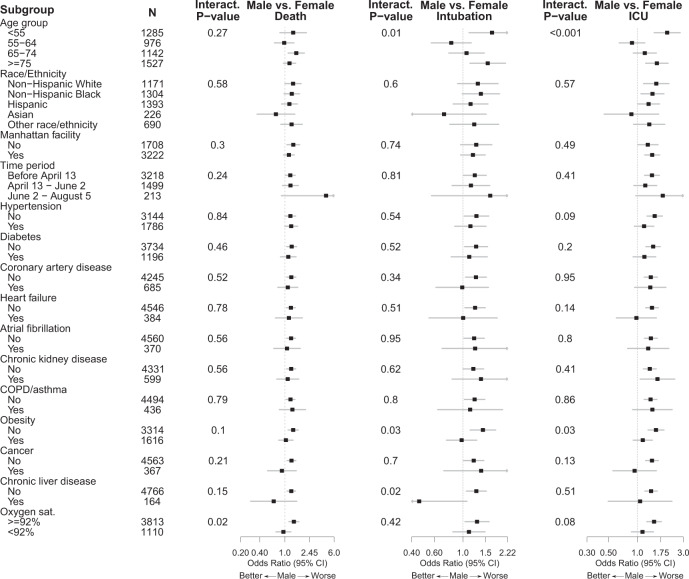
Subgroup analysis for male sex as a predictor of mortality, intubation, or ICU care. The forest plot depicts the odds ratio associated with male sex (versus female sex) within the subgroup specified in a multivariable logistic regression model, including an interaction term between the subgroup variable and sex and adjusting for the other variables listed. The interaction *p* value for sex and each subgroup variable is shown.

Subgroup analysis for predictors of mortality identified a significant interaction between sex and baseline hypoxia (interaction *p* = 0.02) (Fig. 1 and Supplemental Table 5). Among predictors of intubation, there were significant sex interactions for age (interaction *p* = 0.01), obesity (interaction *p* = 0.03), and chronic liver disease (interaction *p* = 0.02). Among predictors of ICU-level care, there were significant sex interactions for age (interaction *p* < 0.001) and obesity (interaction *p* = 0.02); there were also suggestive but non-significant interactions with hypertension (interaction *p* = 0.09) and hypoxia (interaction *p* = 0.08).

**Fig. 2:**
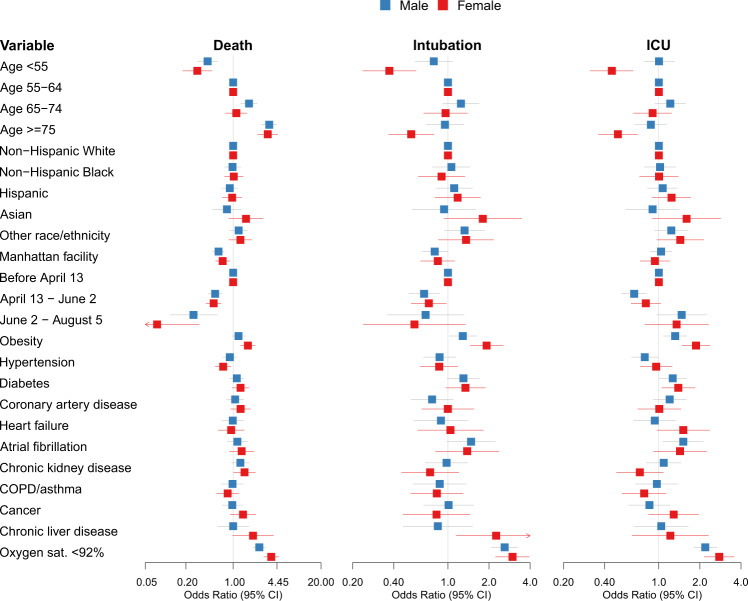
Forest plots depicting sex-stratified multivariable logistic regression models predicting mortality (Male *N* = 2657; Female *N* = 2099), intubation (Male *N* =  2669; Female *N* = 2108), or ICU care (Male *N* = 2669; Female *N* = 2108). The odds ratio associated with each variable is depicted in blue for the male-specific models and in red for the female-specific models.

In sex-stratified models, we observed that while hypoxia was associated with increased mortality in both sexes, the effect size was larger among women (OR 3.67, 95% CI 2.85–4.73) than men (OR 2.44, 95% CI 1.98–3.01) (Fig. 2 and Supplemental Table 6). Similarly, obesity was associated with increased risk of intubation and ICU-level care in both sexes, but had a greater effect on the risk among women (Intubation OR 1.92, 95% CI 1.45–2.54; ICU OR 1.95, 95% CI 1.52–2.49) than men (Intubation OR 1.28, 95% CI 1.02–1.63; ICU OR 1.32, 95% CI 1.08–1.6). Chronic liver disease was associated with a greater risk of intubation in women (OR 2.26, 95% CI 1.15–4.44) than men (OR 0.84, 95% CI 0.47–1.51). Within the youngest (<55) and oldest (≥75) age groups, women had a lower risk of intubation and ICU care than men.

### Validation cohort

To assess the stability of our findings, we assembled a validation cohort consisting of 1645 patients admitted with COVID-19 from August 5, 2020, to January 13, 2021. The validation cohort had a similar age (median 66 vs. 66, Fisher’s exact *p* = 0.3) and sex (56% vs. 55.9% male, Fisher’s exact *p* = 1) distribution as the primary cohort, but had more White patients (29.4% vs. 24.5%, Fisher’s exact *p* = 7.3 × 10^−29^) and had lower rates of comorbidities such as hypertension (30.2% vs. 36.9%, Fisher’s exact *p* = 6.3 × 10^−7^), diabetes (19.6% vs. 24.6%, Fisher’s exact *p* = 2.5 × 10^−5^), and chronic kidney disease (8.9% vs. 12.6%, Fisher’s exact *p* = 4.3 × 10^−5^) (Supplemental Table 7). One notable exception was obesity, which was more common (36.2% vs 32.9%, Fisher’s exact *p* = 0.02) in the validation cohort.

Validation patients were less likely to be febrile (17.6% vs. 19.8%, Fisher’s exact *p* = 0.05), tachycardic (31.8% vs. 40.1%, Fisher’s exact *p* = 9.9 × 10^−14^), hypotensive (2.4% vs. 3.6%, Fisher’s exact *p* = 0.03), or hypoxic (13.5% vs. 22.6%, Fisher’s exact *p* < 0.001) on presentation. Treatment with remdesivir (35% vs. 2.8%, Fisher’s exact *p* = 4.0 × 10^−246^) and dexamethasone (65.8% vs. 3.3%, Fisher’s exact *p* < 0.001) were significantly more common in the validation cohort. Unadjusted outcomes were also better in validation cohort: mortality (10.2% vs. 24.1%, Fisher’s exact *p* = 1.8 × 10^−34^), intensive care (19% vs. 23.4%, Fisher’s exact *p* = 1.7 × 10^−4^), and intubation (9.4% vs. 15%, Fisher’s exact *p* = 3.2 × 10^−9^) (Supplemental Table 7).

Multivariable logistic regression in the validation cohort confirmed male sex as an independent risk factor for in-hospital mortality (OR 1.56, 95% CI 1.05–2.32), intensive care (OR 1.65, 95% CI 1.24–2.2), and intubation (OR 1.72, 95% CI 1.17-2.53) (Supplemental Table 8). Older age (age ≥ 74 vs. 55–64 OR 8.5, 95% CI 4.25–16.9), hypoxia (OR 3.9, 95% CI 2.49–6.1), and chronic kidney disease (OR 2.04, 95% CI 1.13–3.67) were also replicated as independent predictors of mortality. There was no significant interaction between sex and hypoxia with regards to mortality (interaction *p* = 0.94), but there was an interaction between sex and obesity (interaction *p* = 0.03) (Supplemental Fig. 3). Obesity had a greater impact on men’s risk of death (OR 1.82, 95% CI 1.04–3.19) than women’s (OR 0.74, 95% CI 0.39–1.42) (Supplemental Table 9).

## Discussion

Sex differences in prevalence, pathogenesis, and outcomes have been observed across a variety of infectious diseases^27^. In general, men are more susceptible to infection while women mount more vigorous immune responses^28,29^. However, this does not necessarily translate into worse outcomes for men; a 2010 WHO study concluded that influenza outcomes are often worse for women, even though they consistently produce higher antibody titers in response to vaccination^30,31^. Sex differences in immunity may be related to the “dose” of immune-related genes located on the X chromosome, or sex hormonal effects on immune cells^32^.

Observational cohort studies have consistently identified a difference in mortality outcomes between men and women infected with SARS-CoV-2^1–6^. An emerging body of literature has characterized differences in cytokines between men and women with COVID-19^26,32–34^. However, these findings have yet to be translated into effective therapies, indicating that our mechanistic understanding of COVID-19 pathogenesis and sex differences remains limited. To enhance our understanding of the effect of sex on COVID-19 outcomes, we characterized the COVID-19 disease course of hospitalized men and women in a large health-system cohort and used stratified and interaction analyses to identify interactions between sex and other COVID-19 risk factors.

Men in our primary cohort were younger than the women and were less likely to have common comorbidities such as obesity and hypertension, which have been linked to adverse COVID-19 outcomes^20,35^. Despite this apparently favorable clinical profile, men were more likely to present with signs of severe disease, such as hypoxia and lymphopenia^10,36^, and were more likely to be intubated (16.4% vs. 13.2%, Fisher’s exact *p* = 0.002) or receive intensive care (26.7% vs. 20.3%, Fisher’s exact *p* = 1.8 × 10^−7^). After adjusting for demographics and comorbidities, male sex was an independent risk factor for mortality, intubation, and intensive care, in accordance with findings of studies from the USA^2,5^, the UK^6^, Italy^1^, and China^11^. Male sex remained an independent risk factor for adverse outcomes in a validation cohort comprised of patients admitted to our health system at a later phase of the pandemic.

Given this persistent male–female disparity, we reasoned that there might be clinical factors, which preferentially affected men’s or women’s outcomes. By testing interaction terms and using sex-stratified models, we identified hypoxia and obesity as risk factors, which preferentially increased women’s risk of mortality and intubation/intensive care, respectively, in the primary cohort. However, in the validation cohort, the sex specificity of hypoxia did not replicate, while the sex specificity of obesity was reversed.

In the primary cohort, men were more likely to present with hypoxia (24.3% vs. 20.2%, Fisher’s exact *p* = 8.6 × 10^−4^), but the presence of hypoxia only increased men’s risk of death by 2.44 times, compared to an increase of 3.67 times for women (interaction *p* = 0.02). In the validation cohort, hypoxia remained an independent predictor of mortality (OR 3.9, 95% CI 2.49–6.1), but there was no significant interaction between sex and hypoxia (interaction *p* = 0.94).

In a recent study of 6916 patients in Southern California, Tartof et al. reported an interaction between BMI and sex; increasing BMI had a greater impact on men’s mortality risk than women’s^35^. By contrast, we initially found that obesity preferentially increased women’s risk of intubation and intensive care while there was no interaction between sex and obesity with regards to in-hospital mortality. However, the validation cohort showed a significant interaction between sex and obesity (interaction *p* = 0.03) with higher mortality for obese men, in line with the findings of Tartof et al.

There were also significant interactions in the primary analysis between chronic liver disease and sex with regards to intubation, and between age and sex for both intubation and intensive care. Liver disease had a greater impact on women’s risk of intubation than men; however, the number of patients with chronic liver disease in the primary cohort was small (*N* = 164), and this finding requires further evaluation. The interaction analysis for sex and age revealed that men in the youngest and oldest age groups were more likely than women to be intubated or receive intensive care (Fig. 1). These interactions were not significant in the validation cohort, but power was limited due to the reduced rate of intensive care intubation during the validation period.

Differences in the conditions of the pandemic and patient population may account for the differences in sex-specific risk factors between our primary and validation cohorts. The primary cohort consisted of patients admitted during the initial pandemic surge; there were no proven COVID-19 therapies at that time, and hospital capacity was severely taxed. The validation cohort reflects a later period of the pandemic, after the establishment of evidence-based treatment protocols and without the same strains on bed capacity^37–40^. The use of treatments such as remdesivir and dexamethasone was significantly more common in the validation cohort, and outcomes were correspondingly better. The comorbidity profile of patients in the validation cohort was also different. Consequently, it is challenging to compare results from these two cohorts. Nevertheless, the persistent association of male sex with worse outcomes is notable. Sex-specific interactions noted in either cohort should be taken in context.

The strengths of our study include its size, the diversity of the patient population, and two cohorts encompassing the evolution of the pandemic in New York City from initial surge to implementation of evidence-based treatments. Although larger, national-level patient cohorts have been published^6,41^, this Mount Sinai Health System cohort is on par with other health-system and hospital-network-based cohorts from the US and Italy^1–3,5,8^. The detailed clinical information in this cohort empowers us to explicitly address sex differences in mortality risk factors using a rigorous combination of stratification and interaction models. To our knowledge, we are the first to report comprehensive sex-stratified data on clinical features, risk factors, and their association with outcomes.

This study has several limitations. Our dataset did not capture socioeconomic factors nor pre-admission clinical factors such as duration of symptoms; we cannot rule out the possibility that sex differences in seeking or accessing care may have confounded the outcomes. Comorbidity data were extracted from the electronic medical record and were not manually reviewed; their completeness and accuracy depend on data entered by medical providers during routine clinical care. In addition, due to limited exchange of medical data between institutions, comorbidity data may be incomplete for some patients who receive care from multiple health systems. Our results should be considered hypothesis-generating and require confirmation in other large cohorts as well as causal studies to establish mechanisms.

In conclusion, this study provides a detailed characterization of the clinical features and outcomes of men and women hospitalized with COVID-19. It highlights interactions between sex, obesity, and hypoxia with regards to mortality, intubation, and intensive care. These interactions nominate patient subgroups for further study and provide insights that may help explain the sex differences in outcomes of this disease.

## Supplementary information

Supplementary Information

Description of Additional Supplementary Files

Supplementary Data 1

Supplementary Data 2

Reporting Summary

## Data Availability

Data underlying Figs. 1 and 2 are available in Supplementary Data 1 and 2. Other de-identified data is available in accordance with Mount Sinai policy. Requests will be assessed by the Icahn School of Medicine Institutional Data Access Committee (contact via Joy.Dicker@mssm.edu).

## Code availability

R code associated with these analyses is available at https://github.com/Huang-lab/SParCS/tree/master/MSSM_COVID/R.
