# Supplementary material for: Analysis of sex-specific risk factors and clinical outcomes in COVID-19
Source: Commun Med (Lond). 2021 Jun 30;1:3. doi: 10.1038/s43856-021-00006-2 (PMC9053255; doi:10.1038/s43856-021-00006-2)

Analysis of sex-specific risk factors and their association with clinical outcomes in COVID-19

Tomi Jun[1], Sharon Nirenberg[2], Tziopora Weinberger[3], Navya Sharma[4], Elisabet Pujadas[5], Carlos Cordon-Cardo[5], Patricia Kovatch[2], Kuan-lin Huang[3]*

1. Division of Hematology and Medical Oncology, Tisch Cancer Institute, Icahn School of Medicine at Mount Sinai, New York, NY 10029, USA.
2. Scientific Computing, Icahn School of Medicine at Mount Sinai, New York, NY 10029, USA.
3. Department of Genetics and Genomic Sciences, Center for Transformative Disease Modeling, Tisch Cancer Institute, Icahn Institute for Data Science and Genomic Technology, Icahn School of Medicine at Mount Sinai, New York, NY 10029, USA
4. University of California, Santa Barbara, CA, USA.
5. Department of Pathology, Molecular and Cell-Based Medicine, Icahn School of Medicine at Mount Sinai, New York, NY 10029, USA.

Supplemental materials

Supplemental Table 1: Laboratory values at presentation, by sex.

Supplemental Table 2: Treatments received, stratified by ICU status and sex.

Supplemental Table 3: Steroids received, by sex.

Supplemental Table 4: Multivariable logistic regression for in-hospital mortality, ICU-level care, and intubation with oxygen saturation modeled as a continuous variable.

Supplemental Table 5: Subgroup analyses for outcomes of death, intubation, and intensive care. Odds ratios are for male sex relative to female sex.

Supplemental Table 6: Sex-stratified multivariable logistic regression models for death, intubation, and ICU admission.

Supplemental Table 7: Clinical characteristics and outcomes of validation cohort (January 2021) compared to primary cohort (August 2020).

Supplemental Table 8: Multivariable logistic regression for in-hospital mortality, ICU-level care, and intubation using the January 2021 validation cohort.

Supplemental Table 9: Sex-stratified multivariable logistic regression models for death, intubation, and ICU admission using the January 2021 validation cohort.

Supplemental Figure 1: Box plots depicting SARS-CoV-2 viral loads by sex in the Pujadas et al. cohort. The median is represented by the dark line while the lower and upper bounds of the box represent the 25th and 75th percentiles, respectively. The whiskers reflect the maximum and minimum values of the data.

Supplemental Figure 2: Table and forest plot of multivariable logistic regression models predicting mortality, intubation, or ICU care. Odds ratios are adjusted for all variables listed.

Supplemental Figure 3: Subgroup analysis for male sex as a predictor of mortality in the January 2021 validation cohort. Each subgroup is analyzed using an interaction model between sex and the subgroup variable, adjusting for the other variables listed. The odds ratio for male sex within each strata is presented in the table and forest plot. The interaction p-value for sex and each subgroup variable is shown in the table.

Supplemental Table 1: Laboratory values at presentation, by sex.

Variable	Male (N=2757)	Female (N=2173)	P-value	Missing values (%)
White blood cells, 10^3/uL	7.9 (5.7 - 10.9)	7.6 (5.5 - 10.6)	0.1	0
Hemoglobin, g/dL	13.6 (11.9 - 14.8)	12.4 (11 - 13.5)	<0.001	0
Platelets, 10^3/uL	204 (156 - 275)	215 (166 - 279.75)	<0.001	0.01
Neutrophils, 10^3/uL	6.14 (4.1 - 8.98)	5.79 (3.95 - 8.53)	0.005	0.02
Lymphocytes, 10^3/uL	0.87 (0.6 - 1.23)	1.05 (0.7 - 1.51)	<0.001	0.02
Monocytes, 10^3/uL	0.5 (0.34 - 0.73)	0.48 (0.31 - 0.69)	<0.001	0.02
Basophils, 10^3/uL	0.01 (0 - 0.03)	0.02 (0.01 - 0.03)	<0.001	0.03
Eosinophils, 10^3/uL	0.01 (0.01 - 0.04)	0.02 (0.01 - 0.05)	0.005	0.03
Sodium, mmol/L	137 (133 - 140)	138 (135 - 141)	<0.001	0.14
Potassium, mmol/L	4.3 (3.9 - 4.7)	4.1 (3.7 - 4.6)	<0.001	0.05
Chloride, mmol/L	102 (98 - 106)	103 (100 - 107)	<0.001	0.02
Blood urea nitrogen, mg/dL	20 (13 - 37)	17 (11 - 33.25)	<0.001	0.02
Creatinine, mg/dL	1.11 (0.87 - 1.75)	0.9 (0.68 - 1.5)	<0.001	0.02
Aspartate aminotransferase, U/L	46 (30 - 74)	36 (25 - 57)	<0.001	0.13
Alanine aminotransferase, U/L	34 (20 - 58)	24 (16 - 39)	<0.001	0.08
Total bilirubin, mg/dL	0.6 (0.5 - 0.9)	0.5 (0.4 - 0.7)	<0.001	0.08
Albumin, g/dL	3.2 (2.8 - 3.5)	3.1 (2.8 - 3.5)	0.1	0.07
Ferritin, ng/mL	938.5 (456.25 - 2127.75)	459 (209 - 963)	<0.001	0.25
Ferritin, xUpper limit of normal	2.35 (1.14 - 5.32)	3.06 (1.39 - 6.42)	<0.001	0.25
Procalcitonin, ng/mL	0.22 (0.1 - 0.69)	0.15 (0.06 - 0.55)	<0.001	0.28
Lactate dehydrogenase, U/L	438 (324.5 - 597)	388 (293 - 532)	<0.001	0.31
C-reactive protein, mg/L	125.3 (63.61 - 211.2)	88.4 (36.35 - 175.5)	<0.001	0.24
Troponin-I, ng/mL	0.03 (0.02 - 0.11)	0.04 (0.02 - 0.11)	0.4	0.38
Interleukin-1-beta, pg/mL	0.6 (0.4 - 1)	0.6 (0.4 - 0.8)	0.4	0.86
Interleukin-6, pg/mL	78.65 (37.52 - 144.15)	57.85 (30.8 - 121.25)	<0.001	0.67
Interleukin-8, pg/mL	41.3 (26.4 - 67.65)	41.1 (26.47 - 64.6)	0.8	0.81
Tumor necrosis factor-alpha, pg/mL	22.1 (16.5 - 31.9)	21.9 (15.6 - 32.7)	0.6	0.81

Values presented as medians and interquartile ranges; P-values from the Wilcoxon rank-sum test.

Supplemental Table 2: Treatments received, stratified by ICU status and sex.

Cohort	Treatment	Male	Female	P	Total
Overall	TOCILIZUMAB	141 (5.1%)	94 (4.3%)	0.202	235
Overall	REMDESIVIR	86 (3.1%)	53 (2.4%)	0.166	139
Overall	SARILUMAB	26 (0.9%)	5 (0.2%)	0.002	31
Overall	HYDROXYCHLOROQUINE	1862 (67.5%)	1294 (59.5%)	<0.001	3156
Overall	ANAKINRA	3 (0.1%)	1 (0%)	0.635	4
Overall	AZITHROMYCIN	1805 (65.5%)	1265 (58.2%)	<0.001	3070
Overall	CONVALESCENT_PLASMA	179 (6.5%)	136 (6.3%)	0.769	315
Overall	STEROID	801 (29.1%)	542 (24.9%)	0.001	1343
Overall	DEXAMETHASONE	92 (3.3%)	72 (3.3%)	1	164
ICU	TOCILIZUMAB	108 (14.7%)	70 (15.9%)	0.61	178
ICU	REMDESIVIR	34 (4.6%)	20 (4.5%)	1	54
ICU	SARILUMAB	24 (3.3%)	4 (0.9%)	0.01	28
ICU	HYDROXYCHLOROQUINE	530 (72.1%)	297 (67.3%)	0.09	827
ICU	ANAKINRA	3 (0.4%)	1 (0.2%)	1	4
ICU	AZITHROMYCIN	524 (71.3%)	310 (70.3%)	0.74	834
ICU	CONVALESCENT_PLASMA	67 (9.1%)	54 (12.2%)	0.09	121
ICU	STEROID	404 (55%)	255 (57.8%)	0.36	659
ICU	DEXAMETHASONE	44 (6%)	33 (7.5%)	0.33	77
NON-ICU	TOCILIZUMAB	33 (1.6%)	24 (1.4%)	0.59	57
NON-ICU	REMDESIVIR	52 (2.6%)	33 (1.9%)	0.19	85
NON-ICU	SARILUMAB	2 (0.1%)	1 (0.1%)	1	3
NON-ICU	HYDROXYCHLOROQUINE	1332 (65.9%)	997 (57.6%)	<0.001	2329
NON-ICU	AZITHROMYCIN	1281 (63.4%)	955 (55.1%)	<0.001	2236
NON-ICU	CONVALESCENT_PLASMA	112 (5.5%)	82 (4.7%)	0.3	194
NON-ICU	STEROID	397 (19.6%)	287 (16.6%)	0.02	684
NON-ICU	DEXAMETHASONE	48 (2.4%)	39 (2.3%)	0.83	87

Supplemental Table 3: Steroids received, by sex.

Steroid	Male	Female	P	Total
ANY	801 (29.1%)	542 (24.9%)	0.001	1343
PREDNISONE	222 (8.1%)	164 (7.5%)	0.522	386
DEXAMETHASONE	92 (3.3%)	72 (3.3%)	1	164
HYDROCORTISONE	12 (0.4%)	3 (0.1%)	0.07	15
METHYLPREDNISOLONE	639 (23.2%)	428 (19.7%)	0.003	1067

Supplemental Table 4: Multivariable logistic regression for in-hospital mortality, ICU-level care, and intubation with oxygen saturation modeled as a continuous variable.

Variable	Death Adj. OR (95% CI)	P-value	Intubation Adj. OR (95% CI)	P-value	ICU Adj. OR (95% CI)	P-value
Age <55	0.368 (0.277-0.487)	<0.001	0.611 (0.473-0.791)	<0.001	0.763 (0.619-0.94)	0.011
Age 55-64	Ref	-	Ref	-	Ref	-
Age 65-74	1.473 (1.18-1.84)	<0.001	1.143 (0.903-1.45)	0.27	1.087 (0.887-1.33)	0.421
Age >=75	3.32 (2.69-4.12)	<0.001	0.734 (0.573-0.94)	0.01	0.689 (0.558-0.849)	<0.001
Male	1.226 (1.05-1.43)	0.009	1.214 (1.02-1.45)	0.03	1.359 (1.17-1.57)	<0.001
Non-Hispanic White	Ref	-	Ref	-	Ref	-
Non-Hispanic Black	1.025 (0.835-1.26)	0.813	1.015 (0.794-1.3)	0.91	1.044 (0.852-1.28)	0.676
Hispanic	0.928 (0.754-1.14)	0.483	1.13 (0.89-1.43)	0.32	1.132 (0.929-1.38)	0.217
Asian	1.021 (0.705-1.48)	0.911	1.154 (0.759-1.75)	0.5	1.071 (0.75-1.53)	0.705
Other race/ethnicity	1.185 (0.933-1.51)	0.164	1.255 (0.953-1.65)	0.11	1.252 (0.994-1.58)	0.056
Manhattan facility	0.645 (0.554-0.75)	<0.001	0.818 (0.686-0.974)	0.02	0.998 (0.859-1.16)	0.978
Before April 13	Ref	-	Ref	-	Ref	-
April 13 - June 2	0.552 (0.466-0.652)	<0.001	0.712 (0.586-0.866)	<0.001	0.737 (0.628-0.866)	<0.001
June 2 - August 5	0.165 (0.0803-0.338)	<0.001	0.638 (0.378-1.08)	0.09	1.502 (1.08-2.09)	0.015
Obesity	1.387 (1.18-1.63)	<0.001	1.525 (1.27-1.82)	<0.001	1.539 (1.32-1.79)	<0.001
Hypertension	0.817 (0.684-0.976)	0.026	0.876 (0.713-1.08)	0.21	0.862 (0.725-1.02)	0.092
Diabetes	1.178 (0.98-1.42)	0.081	1.28 (1.04-1.58)	0.02	1.289 (1.08-1.54)	0.005
Coronary artery disease	1.192 (0.958-1.48)	0.115	0.879 (0.666-1.16)	0.36	1.145 (0.917-1.43)	0.232
Heart failure	0.989 (0.744-1.32)	0.942	0.989 (0.693-1.41)	0.95	1.175 (0.888-1.55)	0.259
Atrial fibrillation	1.208 (0.923-1.58)	0.169	1.419 (1.02-1.97)	0.04	1.468 (1.12-1.93)	0.006
Chronic kidney disease	1.349 (1.07-1.7)	0.012	0.87 (0.651-1.16)	0.35	0.937 (0.741-1.18)	0.584
COPD/asthma	0.911 (0.705-1.18)	0.478	0.897 (0.657-1.22)	0.49	0.91 (0.706-1.17)	0.467
Cancer	1.082 (0.823-1.42)	0.573	0.882 (0.629-1.24)	0.47	0.976 (0.744-1.28)	0.86
Chronic liver disease	1.251 (0.818-1.91)	0.302	1.231 (0.789-1.92)	0.36	1.074 (0.737-1.57)	0.71
Oxygen saturation	0.934 (0.924-0.944)	<0.001	0.937 (0.927-0.947)	<0.001	0.94 (0.93-0.949)	<0.001

Supplemental Table 5: Subgroup analyses for outcomes of death, intubation, and intensive care. Odds ratios are for male sex relative to female sex.

Variable	N	Death Interact. P-Value	Death M vs. F OR (95% CI)	Intubation Interact. P-Value	Intubation M vs. F OR (95% CI)	ICU Interact. P-Value	ICU M vs. F OR (95% CI)
Age group							
<55	1285	0.27	1.35 (0.84-2.19)	0.01	1.68 (1.12-2.53)	<0.001	2.04 (1.49-2.78)
55-64	976	0.27	0.98 (0.69-1.39)	0.01	0.81 (0.57-1.16)	<0.001	0.88 (0.65-1.19)
65-74	1142	0.27	1.51 (1.13-2.03)	0.01	1.07 (0.78-1.46)	<0.001	1.19 (0.9-1.57)
>=75	1527	0.27	1.18 (0.95-1.48)	0.01	1.56 (1.14-2.14)	<0.001	1.59 (1.22-2.08)
Race/Ethnicity							
Non-Hispanic White	1171	0.58	1.35 (1-1.81)	0.6	1.31 (0.91-1.87)	0.57	1.57 (1.16-2.12)
Non-Hispanic Black	1304	0.58	1.29 (0.97-1.7)	0.6	1.38 (0.99-1.92)	0.57	1.43 (1.09-1.88)
Hispanic	1393	0.58	1.18 (0.89-1.57)	0.6	1.15 (0.84-1.56)	0.57	1.31 (1.01-1.69)
Asian	226	0.58	0.72 (0.36-1.43)	0.6	0.71 (0.34-1.51)	0.57	0.87 (0.45-1.66)
Other race/ethnicity	690	0.58	1.27 (0.86-1.88)	0.6	1.22 (0.79-1.89)	0.57	1.33 (0.92-1.92)
Manhattan facility							
No	1708	0.3	1.36 (1.07-1.71)	0.74	1.27 (0.96-1.68)	0.49	1.28 (1.01-1.64)
Yes	3222	0.3	1.16 (0.95-1.41)	0.74	1.2 (0.96-1.49)	0.49	1.42 (1.19-1.7)
Time period							
Before April 13	3218	0.24	1.22 (1.02-1.46)	0.81	1.24 (1.01-1.52)	0.41	1.42 (1.19-1.69)
April 13 - June 2	1499	0.24	1.23 (0.92-1.63)	0.81	1.15 (0.82-1.61)	0.41	1.21 (0.92-1.59)
June 2 - August 5	213	0.24	4.48 (0.88-22.77)	0.81	1.63 (0.57-4.65)	0.41	1.83 (0.96-3.49)
Hypertension							
No	3144	0.84	1.22 (1.01-1.48)	0.54	1.27 (1.02-1.58)	0.09	1.51 (1.26-1.82)
Yes	1786	0.84	1.26 (1-1.59)	0.54	1.14 (0.86-1.51)	0.09	1.17 (0.93-1.48)
Diabetes							
No	3734	0.46	1.28 (1.07-1.53)	0.52	1.27 (1.03-1.55)	0.2	1.46 (1.23-1.73)
Yes	1196	0.46	1.13 (0.85-1.5)	0.52	1.12 (0.81-1.54)	0.2	1.18 (0.9-1.55)
Coronary artery disease							

No	4245	0.52	1.26 (1.07-1.49)	0.34	1.26 (1.05-1.52)	0.95	1.38 (1.18-1.61)
Yes	685	0.52	1.11 (0.77-1.6)	0.34	0.99 (0.62-1.58)	0.95	1.36 (0.94-1.97)
Heart failure							
No	4546	0.78	1.24 (1.06-1.46)	0.51	1.24 (1.04-1.49)	0.14	1.42 (1.22-1.65)
Yes	384	0.78	1.16 (0.71-1.89)	0.51	1 (0.55-1.84)	0.14	0.97 (0.61-1.57)
Atrial fibrillation							
No	4560	0.56	1.25 (1.07-1.47)	0.95	1.22 (1.02-1.46)	0.8	1.38 (1.19-1.61)
Yes	370	0.56	1.08 (0.67-1.75)	0.95	1.24 (0.69-2.24)	0.8	1.29 (0.79-2.11)
Chronic kidney disease							
No	4331	0.56	1.26 (1.07-1.48)	0.62	1.21 (1-1.45)	0.41	1.34 (1.15-1.57)
Yes	599	0.56	1.11 (0.74-1.65)	0.62	1.38 (0.82-2.31)	0.41	1.61 (1.07-2.44)
COPD/asthma							
No	4494	0.79	1.23 (1.05-1.44)	0.8	1.23 (1.03-1.48)	0.86	1.37 (1.17-1.59)
Yes	436	0.79	1.32 (0.82-2.12)	0.8	1.14 (0.64-2.03)	0.86	1.43 (0.89-2.29)
Obesity							
No	3314	0.1	1.35 (1.12-1.63)	0.03	1.43 (1.14-1.79)	0.03	1.56 (1.29-1.88)
Yes	1616	0.1	1.04 (0.8-1.34)	0.03	0.98 (0.75-1.28)	0.03	1.14 (0.9-1.43)
Cancer							
No	4563	0.21	1.27 (1.09-1.49)	0.7	1.21 (1.01-1.45)	0.13	1.42 (1.22-1.65)
Yes	367	0.21	0.9 (0.53-1.51)	0.7	1.38 (0.71-2.69)	0.13	0.93 (0.56-1.57)
Chronic liver disease							
No	4766	0.15	1.26 (1.08-1.47)	0.02	1.27 (1.06-1.52)	0.51	1.39 (1.2-1.61)
Yes	164	0.15	0.67 (0.29-1.54)	0.02	0.46 (0.19-1.07)	0.51	1.07 (0.5-2.28)
Oxygen sat.							
>=92%	3813	0.02	1.39 (1.16-1.66)	0.42	1.29 (1.04-1.59)	0.08	1.49 (1.25-1.77)
<92%	1110	0.02	0.95 (0.72-1.24)	0.42	1.11 (0.84-1.48)	0.08	1.13 (0.87-1.47)

Supplemental Table 6: Sex-stratified multivariable logistic regression models for death, intubation, and ICU admission.

Variable	Death, Male Adj. OR (95% CI)	Death, Female Adj. OR (95% CI)	Intubation, Male Adj. OR (95% CI)	Intubation, Female Adj. OR (95% CI)	ICU, Male Adj. OR (95% CI)	ICU, Female Adj. OR (95% CI)
Age <55	0.42 (0.3-0.59)	0.3 (0.18-0.49)	0.79 (0.58-1.08)	0.37 (0.24-0.58)	1 (0.78-1.3)	0.45 (0.31-0.65)
Age 55-64	Ref	Ref	Ref	Ref	Ref	Ref
Age 65-74	1.72 (1.3-2.27)	1.12 (0.77-1.61)	1.24 (0.92-1.69)	0.96 (0.66-1.39)	1.21 (0.94-1.57)	0.9 (0.65-1.24)
Age >=75	3.42 (2.59-4.49)	3.25 (2.3-4.56)	0.95 (0.69-1.31)	0.54 (0.37-0.79)	0.87 (0.67-1.14)	0.5 (0.36-0.7)
Non-Hispanic White	Ref	Ref	Ref	Ref	Ref	Ref
Non-Hispanic Black	0.98 (0.75-1.29)	1.02 (0.74-1.41)	1.06 (0.77-1.45)	0.9 (0.61-1.33)	1.02 (0.78-1.33)	1 (0.72-1.39)
Hispanic	0.89 (0.68-1.17)	0.97 (0.7-1.34)	1.11 (0.82-1.51)	1.18 (0.8-1.73)	1.07 (0.83-1.37)	1.24 (0.9-1.71)
Asian	0.81 (0.5-1.31)	1.55 (0.86-2.77)	0.94 (0.54-1.61)	1.8 (0.94-3.46)	0.9 (0.57-1.41)	1.6 (0.9-2.84)
Other race/ethnicity	1.21 (0.89-1.64)	1.28 (0.86-1.9)	1.32 (0.94-1.85)	1.36 (0.85-2.16)	1.23 (0.93-1.64)	1.44 (0.96-2.13)
Manhattan facility	0.61 (0.5-0.74)	0.7 (0.55-0.89)	0.8 (0.64-1)	0.84 (0.63-1.12)	1.04 (0.86-1.25)	0.94 (0.73-1.2)
Before April 13	Ref	Ref	Ref	Ref	Ref	Ref
April 13 - June 2	0.54 (0.43-0.68)	0.52 (0.4-0.66)	0.67 (0.52-0.86)	0.72 (0.54-0.98)	0.66 (0.53-0.81)	0.8 (0.63-1.03)
June 2 - August 5	0.26 (0.12-0.58)	0.07 (0.02-0.31)	0.69 (0.36-1.31)	0.57 (0.24-1.34)	1.47 (0.96-2.24)	1.35 (0.79-2.31)
Obesity	1.2 (0.96-1.5)	1.66 (1.29-2.13)	1.28 (1.02-1.63)	1.92 (1.45-2.54)	1.32 (1.08-1.6)	1.87 (1.48-2.37)
Hypertension	0.89 (0.71-1.13)	0.71 (0.54-0.93)	0.87 (0.66-1.14)	0.86 (0.63-1.18)	0.79 (0.63-0.99)	0.96 (0.73-1.25)
Diabetes	1.13 (0.89-1.44)	1.29 (0.96-1.71)	1.3 (0.99-1.71)	1.34 (0.96-1.88)	1.26 (1-1.59)	1.39 (1.05-1.84)
Coronary artery disease	1.07 (0.8-1.43)	1.29 (0.92-1.81)	0.76 (0.53-1.09)	1 (0.64-1.55)	1.2 (0.91-1.59)	1.01 (0.7-1.45)
Heart failure	0.99 (0.68-1.44)	0.93 (0.6-1.46)	0.89 (0.56-1.41)	1.04 (0.6-1.81)	0.93 (0.65-1.34)	1.51 (0.97-2.36)
Atrial fibrillation	1.16 (0.82-1.65)	1.34 (0.88-2.04)	1.48 (0.98-2.22)	1.38 (0.81-2.36)	1.51 (1.07-2.12)	1.43 (0.91-2.23)
Chronic kidney disease	1.28 (0.95-1.73)	1.48 (1.02-2.14)	0.98 (0.68-1.4)	0.74 (0.46-1.2)	1.09 (0.81-1.45)	0.72 (0.49-1.08)
COPD/asthma	0.98 (0.68-1.41)	0.83 (0.57-1.21)	0.87 (0.56-1.36)	0.83 (0.53-1.29)	0.97 (0.68-1.38)	0.78 (0.54-1.13)
Cancer	0.97 (0.69-1.38)	1.4 (0.91-2.16)	1.01 (0.66-1.54)	0.82 (0.47-1.45)	0.85 (0.6-1.21)	1.28 (0.84-1.96)
Chronic liver disease	1 (0.59-1.7)	1.97 (0.98-3.95)	0.84 (0.47-1.51)	2.26 (1.15-4.44)	1.04 (0.66-1.64)	1.21 (0.64-2.31)
Oxygen sat. <92%	2.44 (1.98-3.01)	3.67 (2.85-4.73)	2.6 (2.08-3.25)	2.97 (2.23-3.94)	2.19 (1.8-2.67)	2.77 (2.15-3.57)

Supplemental Table 7: Clinical characteristics and outcomes of validation cohort (January 2021) compared to primary cohort (August 2020)

	August 2020 (N=4930)	January 2021 (N=1645)	P-value
Age (yrs)	66 (54 - 78)	66 (52 - 78)	0.3
Age <55	1281 (26%)	466 (28.3%)	0.07
Age 55-64	975 (19.8%)	303 (18.4%)	0.2
Age 65-74	1142 (23.2%)	370 (22.5%)	0.6
Age >=75	1523 (30.9%)	506 (30.8%)	0.9
Male	2753 (55.9%)	921 (56%)	1
Asian	229 (4.8%)	85 (5.5%)	0.3
Hispanic	1404 (29.4%)	356 (22.8%)	<0.001
Non-Hispanic Black	1314 (27.5%)	248 (15.9%)	<0.001
Non-Hispanic White	1171 (24.5%)	615 (39.4%)	<0.001
Other race/ethnicity	661 (13.8%)	255 (16.4%)	0.01
Manhattan treatment facility	3217 (65.4%)	1223 (74.3%)	<0.001
Current/former smoker	1320 (33.2%)	440 (30.7%)	0.09
Body mass index (kg/m2)	27.5 (23.91 - 32.32)	27.81 (24 - 32.24)	0.7
Hypertension	1815 (36.9%)	496 (30.2%)	<0.001
Diabetes	1211 (24.6%)	322 (19.6%)	<0.001
Coronary artery disease	703 (14.3%)	207 (12.6%)	0.09
Heart failure	410 (8.3%)	111 (6.7%)	0.04
Atrial fibrillation	378 (7.7%)	104 (6.3%)	0.07
Chronic kidney disease	618 (12.6%)	146 (8.9%)	<0.001
COPD/asthma	440 (8.9%)	133 (8.1%)	0.3
Obesity	1620 (32.9%)	595 (36.2%)	0.02
Cancer	378 (7.7%)	136 (8.3%)	0.5
Chronic liver disease	167 (3.4%)	51 (3.1%)	0.6
HIV	86 (1.7%)	20 (1.2%)	0.2
Temperature (°F)	98.6 (98 - 99.9)	98.6 (97.9 - 99.7)	<0.001
Fever	976 (19.8%)	290 (17.6%)	0.05
Heart rate (bpm)	96 (83 - 110)	91 (79 - 105)	<0.001
Tachycardia	1971 (40.1%)	523 (31.8%)	<0.001
Systolic blood pressure (mmHg)	129 (115 - 145)	131 (116 - 145)	0.03
Hypotension	176 (3.6%)	40 (2.4%)	0.03
Respiratory rate (bpm)	20 (18 - 22)	20 (18 - 20)	<0.001
Tachypnea	722 (14.7%)	153 (9.3%)	<0.001
Oxygen saturation (%)	95 (92 - 98)	96 (94 - 98)	<0.001
Oxygen sat. <92%	1110 (22.6%)	222 (13.5%)	<0.001
Treated with remdesivir	139 (2.8%)	576 (35%)	<0.001
Treated with steroids	1341 (27.3%)	1190 (72.3%)	<0.001
Treated with dexamethasone	163 (3.3%)	1082 (65.8%)	<0.001
Invasive ventilation	737 (15%)	154 (9.4%)	<0.001
Intensive care	1151 (23.4%)	312 (19%)	<0.001

	In-hospital death	1186 (24.1%)	150 (10.2%)	<0.001

Supplemental Table 8: Multivariable logistic regression for in-hospital mortality, ICU-level care, and intubation using the January 2021 validation cohort.

	In-hospital Mortality		ICU-level care		Intubation	
	Adj. OR (95% CI)	P-value	Adj. OR (95% CI)	P-value	Adj. OR (95% CI)	P-value
Age <55	0.552 (0.214-1.43)	0.219	0.667 (0.425-1.05)	0.079	0.85 (0.43-1.68)	0.64
Age 55-64	Ref	-	Ref	-	Ref	-
Age 65-74	3.19 (1.55-6.6)	0.002	1.902 (1.26-2.88)	0.002	3.034 (1.67-5.49)	<0.001
Age >=75	8.499 (4.25-16.9)	<0.001	1.87 (1.23-2.83)	0.003	2.829 (1.55-5.15)	<0.001
Male	1.559 (1.05-2.32)	0.029	1.652 (1.24-2.2)	<0.001	1.719 (1.17-2.53)	0.006
Non-Hispanic White	Ref	-	Ref	-	Ref	-
Non-Hispanic Black	0.607 (0.309-1.19)	0.146	0.703 (0.453-1.09)	0.115	0.61 (0.321-1.16)	0.133
Hispanic	1.057 (0.633-1.77)	0.832	0.803 (0.555-1.16)	0.245	0.925 (0.568-1.51)	0.753
Asian	0.927 (0.352-2.44)	0.878	1.068 (0.586-1.95)	0.831	1.203 (0.56-2.58)	0.635
Other race/ethnicity	1.996 (1.2-3.32)	0.008	1.068 (0.73-1.56)	0.734	1.33 (0.817-2.16)	0.252
Manhattan facility	0.681 (0.456-1.02)	0.06	1.203 (0.881-1.64)	0.245	0.895 (0.603-1.33)	0.582
Obesity	1.228 (0.812-1.86)	0.331	1.197 (0.896-1.6)	0.224	1.405 (0.957-2.06)	0.082
Hypertension	0.842 (0.529-1.34)	0.467	0.707 (0.494-1.01)	0.057	0.768 (0.484-1.22)	0.262
Diabetes	1.571 (0.963-2.56)	0.07	1.322 (0.901-1.94)	0.153	1.344 (0.819-2.21)	0.241
Coronary artery disease	1.145 (0.683-1.92)	0.608	0.78 (0.504-1.21)	0.266	0.727 (0.409-1.29)	0.277
Heart failure	1.32 (0.673-2.59)	0.42	1.089 (0.622-1.91)	0.765	0.938 (0.443-1.99)	0.868
Atrial fibrillation	1.443 (0.783-2.66)	0.24	1 (0.579-1.73)	1	1.054 (0.531-2.09)	0.88
Chronic kidney disease	2.038 (1.13-3.67)	0.018	1.589 (0.974-2.59)	0.064	1.567 (0.84-2.92)	0.158
COPD/asthma	0.726 (0.368-1.43)	0.355	0.77 (0.452-1.31)	0.335	0.819 (0.411-1.63)	0.569
Cancer	0.885 (0.446-1.75)	0.726	0.714 (0.422-1.21)	0.209	0.936 (0.481-1.82)	0.844
Chronic liver disease	1.478 (0.52-4.2)	0.463	1.202 (0.573-2.52)	0.627	1.302 (0.498-3.41)	0.59
Oxygen sat. <92%	3.896 (2.49-6.1)	<0.001	3.819 (2.77-5.31)	<0.001	4.055 (2.75-6.03)	<0.001

Supplemental Table 9: Sex-stratified multivariable logistic regression for in-hospital mortality, ICU-level are, and intubation using the January 2021 validation cohort.

	In-hospital Mortality		ICU-level care		Intubation	
	Male<br>Adj. OR (95% CI)	Female<br>Adj. OR (95% CI)	Male<br>Adj. OR (95% CI)	Female<br>Adj. OR (95% CI)	Male<br>Adj. OR (95% CI)	Female<br>Adj. OR (95% CI)
Age <55	0.536 (0.187-1.53)	0.525 (0.0447-6.15)	0.674 (0.397-1.15)	0.546 (0.215-1.39)	0.789 (0.361-1.72)	0.928 (0.215-4)
Age 55-64	Ref	Ref	Ref	Ref	Ref	Ref
Age 65-74	2.625 (1.12-6.18)	7.029 (1.45-33.9)	1.575 (0.944-2.63)	2.746 (1.31-5.81)	2.512 (1.23-5.12)	4.855 (1.52-15.5)
Age >=75	7.925 (3.52-18)	14.013 (3.11-63.6)	1.837 (1.09-3.1)	1.933 (0.935-4)	2.945 (1.44-6.1)	2.915 (0.911-9.25)
Non-Hispanic White	Ref	Ref	Ref	Ref	Ref	Ref
Non-Hispanic Black	0.611 (0.242-1.55)	0.562 (0.202-1.56)	0.658 (0.361-1.2)	0.773 (0.399-1.5)	0.589 (0.246-1.41)	0.651 (0.242-1.75)
Hispanic	0.97 (0.485-1.94)	1.106 (0.498-2.46)	0.862 (0.539-1.38)	0.68 (0.367-1.26)	0.969 (0.525-1.79)	0.82 (0.357-1.88)
Asian	1.041 (0.337-3.21)	0.713 (0.0807-6.3)	1.27 (0.634-2.55)	0.708 (0.188-2.67)	1.266 (0.525-3.06)	1.239 (0.246-6.24)
Other race/ethnicity	1.921 (1.01-3.67)	1.904 (0.8-4.53)	1.081 (0.674-1.73)	0.921 (0.468-1.81)	1.218 (0.668-2.22)	1.388 (0.578-3.34)
Manhattan facility	0.727 (0.426-1.24)	0.563 (0.29-1.09)	1.121 (0.758-1.66)	1.365 (0.792-2.35)	0.82 (0.503-1.34)	1.057 (0.511-2.19)
Obesity	1.822 (1.04-3.19)	0.742 (0.388-1.42)	1.332 (0.914-1.94)	0.928 (0.578-1.49)	1.565 (0.955-2.56)	1.083 (0.573-2.05)
Hypertension	0.829 (0.45-1.53)	0.859 (0.4-1.84)	0.492 (0.306-0.792)	1.137 (0.634-2.04)	0.567 (0.309-1.04)	1.241 (0.572-2.69)
Diabetes	1.745 (0.912-3.34)	1.51 (0.679-3.36)	1.539 (0.944-2.51)	1.078 (0.571-2.03)	1.669 (0.897-3.11)	0.918 (0.402-2.1)
Coronary artery disease	1.564 (0.825-2.96)	0.538 (0.205-1.42)	0.961 (0.562-1.64)	0.494 (0.222-1.1)	0.711 (0.346-1.46)	0.74 (0.275-1.99)
Heart failure	0.731 (0.273-1.96)	2.974 (1.1-8.07)	0.76 (0.348-1.66)	1.65 (0.695-3.92)	0.831 (0.3-2.3)	1.064 (0.326-3.47)
Atrial fibrillation	1.669 (0.716-3.89)	1.359 (0.517-3.57)	0.963 (0.47-1.97)	1.012 (0.408-2.51)	1.115 (0.471-2.64)	0.964 (0.282-3.3)
Chronic kidney disease	2.307 (1.03-5.16)	2.243 (0.896-5.62)	1.6 (0.829-3.09)	1.902 (0.897-4.04)	1.196 (0.501-2.86)	2.716 (1.08-6.83)
COPD/asthma	0.574 (0.217-1.52)	1.012 (0.365-2.8)	0.672 (0.311-1.45)	0.78 (0.36-1.69)	0.587 (0.208-1.66)	1.036 (0.39-2.76)
Cancer	1.29 (0.562-2.97)	0.369 (0.0948-1.44)	0.631 (0.312-1.28)	0.742 (0.329-1.67)	1.127 (0.495-2.57)	0.643 (0.201-2.05)
Chronic liver disease	1.459 (0.348-6.11)	1.954 (0.395-9.67)	0.967 (0.339-2.76)	1.978 (0.659-5.93)	0.747 (0.157-3.55)	2.214 (0.582-8.44)
Oxygen sat. <92%	4.179 (2.34-7.51)	3.706 (1.77-7.75)	3.525 (2.35-5.33)	4.179 (2.4-7.32)	4.015 (2.49-6.52)	3.935 (1.95-8.01)

Supplemental Figure 1: Box plots depicting SARS-CoV-2 viral loads by sex in the Pujadas et al. cohort. The median is represented by the dark line while the lower and up per bounds of the box represent the 25 th and 75th percentiles, respectively. The whiskers reflect the maximum and minimum values of the data.

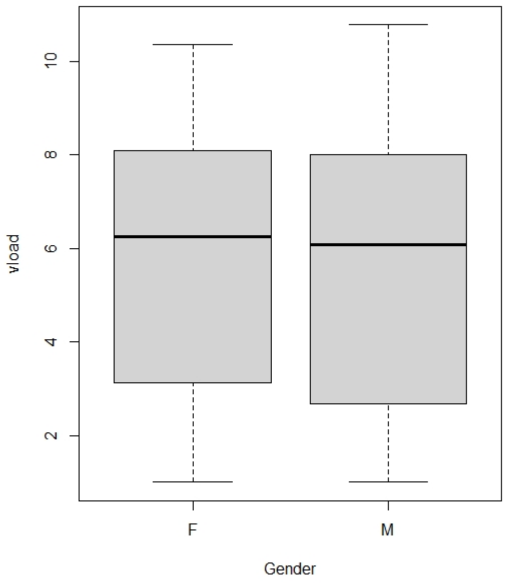

Supplemental Figure 2: Table and forest plot of multivariable logistic regression models predicting mortality, intubation, or ICU care. Odds ratios are adjusted for all variables listed.

Variable	Death OR (95% CI)	Intubation OR (95% CI)	ICU OR (95% CI)
Age <55	0.37 (0.28−0.49)	0.61 (0.47−0.79)	0.76 (0.61−0.93)
Age 55−64	Ref	Ref	Ref
Age 65−74	1.47 (1.18−1.84)	1.15 (0.91−1.45)	1.09 (0.89−1.34)
Age >=75	3.35 (2.71−4.15)	0.76 (0.6−0.97)	0.71 (0.58−0.87)
Male	1.24 (1.06−1.44)	1.22 (1.03−1.46)	1.37 (1.19−1.59)
Non−Hispanic White	Ref	Ref	Ref
Non−Hispanic Black	1.02 (0.83−1.25)	1.02 (0.8−1.31)	1.04 (0.85−1.28)
Hispanic	0.95 (0.78−1.17)	1.18 (0.93−1.49)	1.16 (0.96−1.41)
Asian	1.04 (0.72−1.5)	1.19 (0.78−1.8)	1.09 (0.77−1.56)
Other race/ethnicity	1.25 (0.99−1.59)	1.35 (1.03−1.77)	1.32 (1.05−1.66)
Manhattan facility	0.65 (0.56−0.75)	0.81 (0.69−0.97)	0.99 (0.86−1.15)
Before April 13	Ref	Ref	Ref
April 13 − June 2	0.54 (0.45−0.63)	0.69 (0.57−0.84)	0.72 (0.61−0.84)
June 2 − August 5	0.17 (0.09−0.34)	0.62 (0.37−1.04)	1.42 (1.02−1.97)
Obesity	1.38 (1.17−1.62)	1.51 (1.26−1.81)	1.53 (1.32−1.78)
Hypertension	0.81 (0.68−0.97)	0.87 (0.71−1.07)	0.86 (0.72−1.02)
Diabetes	1.2 (0.99−1.44)	1.3 (1.06−1.61)	1.3 (1.09−1.55)
Coronary artery disease	1.16 (0.93−1.45)	0.85 (0.64−1.12)	1.11 (0.89−1.38)
Heart failure	0.97 (0.73−1.29)	0.96 (0.68−1.37)	1.15 (0.87−1.52)
Atrial fibrillation	1.22 (0.93−1.59)	1.42 (1.03−1.96)	1.46 (1.11−1.91)
Chronic kidney disease	1.36 (1.08−1.72)	0.89 (0.67−1.19)	0.94 (0.75−1.19)
COPD/asthma	0.91 (0.7−1.17)	0.88 (0.65−1.2)	0.9 (0.7−1.17)
Cancer	1.12 (0.86−1.47)	0.93 (0.66−1.29)	1 (0.76−1.31)
Chronic liver disease	1.22 (0.8−1.87)	1.21 (0.78−1.87)	1.05 (0.72−1.52)
Oxygen sat. <92%	2.86 (2.43−3.35)	2.71 (2.28−3.23)	2.41 (2.06−2.81)

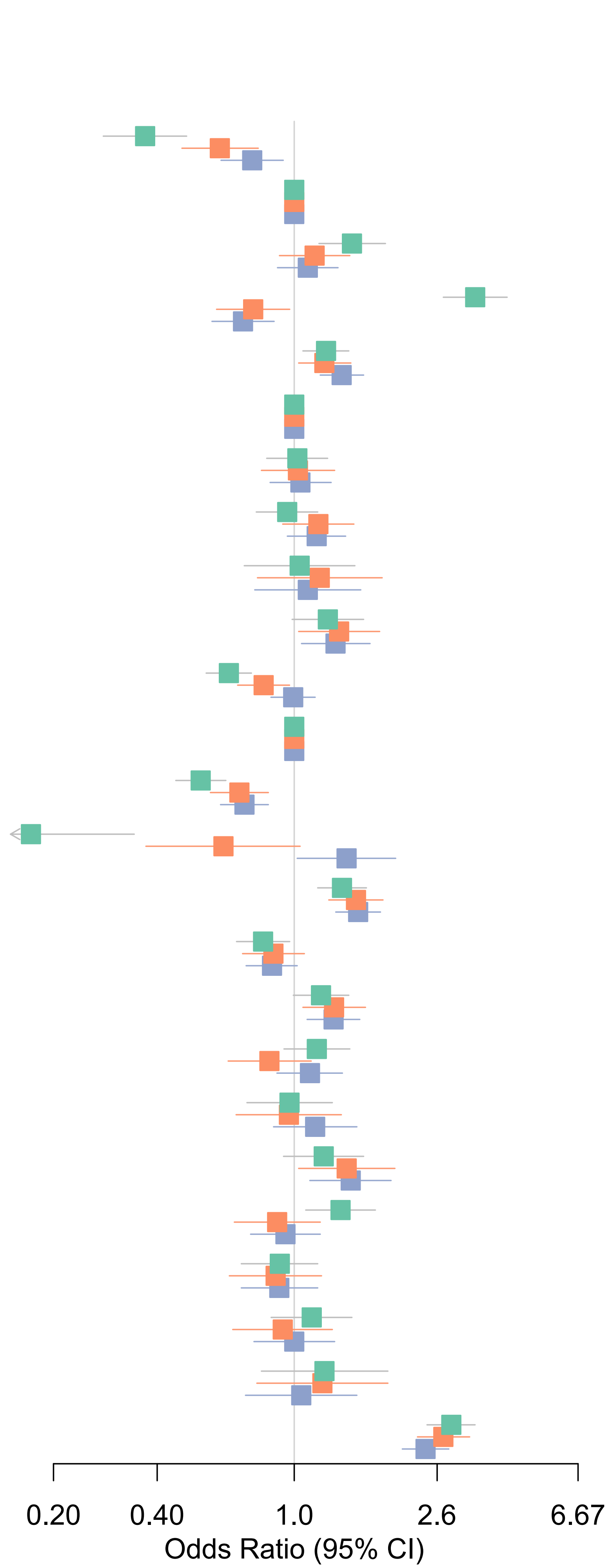

Supplemental Figure 3: Subgroup analysis for male sex as a predictor of mortality in the January 2021 validation cohort. Each subgroup is analyzed using an interaction model between sex and the subgroup variable, adjusting for the other variables listed. The odds ratio for male sex within each strata is presented in the table and forest plot. The interaction p-value for sex and each subgroup variable is shown in the table.

Subgroup	N	Odds ratio (95% CI)	Interact. P−value
Age group			
<55	466	3.1 (0.37−25.77)	0.63
55−64	303	2.92 (0.6−14.14)	0.63
65−74	370	1.15 (0.53−2.51)	0.63
>=75	506	1.55 (0.94−2.56)	0.63
Race/Ethnicity			
Non−Hispanic White	615	1.62 (0.87−3.01)	1
Non−Hispanic Black	248	1.61 (0.49−5.31)	1
Hispanic	356	1.38 (0.63−3.05)	1
Asian	85	2.11 (0.21−21.28)	1
Other race/ethnicity	255	1.57 (0.68−3.63)	1
Manhattan facility			
No	422	1.32 (0.69−2.51)	0.52
Yes	1223	1.72 (1.04−2.84)	0.52
Hypertension			
No	1149	1.45 (0.86−2.43)	0.66
Yes	496	1.73 (0.94−3.17)	0.66
Diabetes			
No	1323	1.45 (0.9−2.32)	0.56
Yes	322	1.86 (0.91−3.81)	0.56
Coronary artery disease			
No	1438	1.38 (0.89−2.14)	0.18
Yes	207	2.71 (1.07−6.84)	0.18
Heart failure			
No	1534	1.71 (1.12−2.61)	0.21
Yes	111	0.79 (0.25−2.47)	0.21
Atrial fibrillation			
No	1541	1.55 (1.01−2.37)	0.94
Yes	104	1.62 (0.55−4.71)	0.94
Chronic kidney disease			
No	1499	1.51 (0.98−2.32)	0.69
Yes	146	1.88 (0.7−5.06)	0.69
COPD/asthma			
No	1512	1.61 (1.06−2.45)	0.58
Yes	133	1.09 (0.3−4.02)	0.58
Obesity			
No	1050	1.14 (0.71−1.85)	0.03
Yes	595	2.85 (1.44−5.64)	0.03
Cancer			
No	1509	1.44 (0.95−2.17)	0.15
Yes	136	4.22 (0.96−18.53)	0.15
Chronic liver disease			
No	1594	1.55 (1.03−2.32)	0.83
Yes	51	1.94 (0.27−13.96)	0.83
Oxygen sat.			
>=92%	1419	1.57 (1−2.46)	0.94
<92%	222	1.52 (0.68−3.4)	0.94

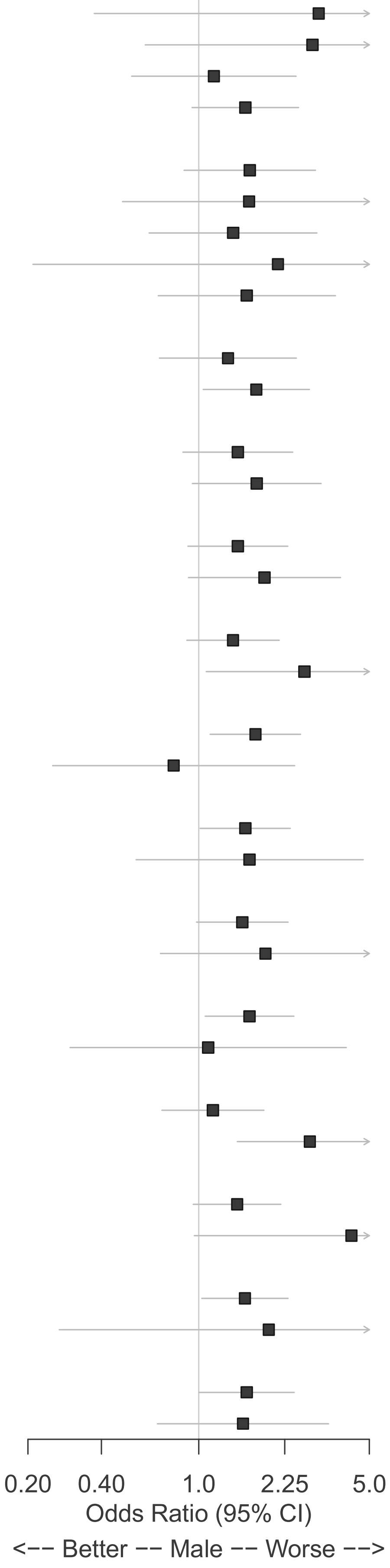

Supplement: Supplementary file 1 — Supplementary Information [file 43856_2021_6_MOESM1_ESM.pdf]
