# Supplementary material for: Analysis of sex-specific risk factors and clinical outcomes in COVID-19
Source: Commun Med (Lond). 2021 Jun 30;1:3. doi: 10.1038/s43856-021-00006-2 (PMC9053255; doi:10.1038/s43856-021-00006-2)
Supplement: Supplementary file 2 — Description of Additional Supplementary Files [file 43856_2021_6_MOESM2_ESM.pdf]

Description of Additional Supplementary Files

File Name: Supplementary Data 1

Description: Data underlying Figure 1

File Name: Supplementary Data 2

Description: Data underlying Figure 2
